# Customer Retention in the Philippine Food Sector: Health Measures, Market Access, and Strategic Adaptation After the COVID-19 Pandemic

**DOI:** 10.3390/foods14142535

**Published:** 2025-07-19

**Authors:** Ma. Janice J. Gumasing

**Affiliations:** Department of Industrial and Systems Engineering, Gokongwei College of Engineering, De La Salle University, 2401 Taft Ave., Manila 1004, Philippines; ma.janice.gumasing@dlsu.edu.ph

**Keywords:** customer retention, casual dining restaurants, food quality, health measures, PLS-SEM

## Abstract

This study investigates the critical determinants of customer retention in casual dining restaurants within the context of the post-pandemic “new normal.” Anchored in service quality and consumer behavior theories, the research examines the influences of food quality, health measures, perceived price, brand image, ambiance, and location on customer decision making. Using Partial Least Squares Structural Equation Modeling (PLS-SEM), data from 336 respondents in the National Capital Region, Philippines were analyzed to assess the relationships among these variables and their effects on restaurant selection and customer retention. The results reveal that food quality (β = 0.698, *p* < 0.05) exerts the strongest influence on restaurant selection, followed by health measures (β = 0.477, *p* = 0.001), perceived price (β = 0.378, *p* < 0.02), and brand image (β = 0.341, *p* < 0.035). Furthermore, health measures (β = 0.436, *p* = 0.002) and restaurant selection (β = 0.475, *p* < 0.05) significantly enhance customer retention, while ambiance and location were not found to be significant predictors. These findings offer theoretical contributions to the service quality and consumer trust literature and provide practical and policy-relevant insights for food establishments adapting to health-driven consumer expectations. The study highlights the need for the strategic integration of safety protocols, pricing value, and brand positioning to foster long-term loyalty and resilience in the evolving food service market.

## 1. Introduction

Customer retention remains a core strategic priority in the food service industry, particularly as businesses continue to adapt to the changes brought about by the COVID-19 pandemic. Retention, defined as a business’s ability to maintain its customer base over time, has direct impacts on profitability, operational stability, and long-term brand equity [1].

In the Philippine context, casual dining is especially popular among urban consumers, notably students and young professionals, due to its balance of affordability, accessibility, and relaxed social environment. Filipino diners also place high value on warm interpersonal service, hospitality, and family-oriented experiences, elements that strengthen brand loyalty and increase the likelihood of repeat visits [2]. The effects of the pandemic were strongly pronounced in highly urbanized areas like Metro Manila, where prolonged lockdowns and rapid digital adoption significantly reshaped dining habits [3]. These cultural and socio-economic dynamics highlight the importance of factors such as food quality, safety, and price sensitivity in influencing consumer decisions in the Philippine casual dining sector.

In a “post COVID-19 pandemic,” context, however, customer retention cannot be understood through pre-crisis models alone. The pandemic disrupted consumer routines, redefined value perceptions, and elevated health and safety as dominant concerns in decision making [4,5]. According to Gupta and Mukherjee [6], COVID-19 catalyzed long-term behavioral changes in consumer habits, including greater risk aversion and demand for contactless experiences, as well as shifting loyalty patterns. As consumers reevaluated their dining choices based on new criteria, such as sanitation protocols, delivery reliability, and health-conscious offerings, the traditional determinants of customer retention have evolved [7]. These changes are not temporary, but are now embedded in the “post COVID-19 pandemic” context of food service, where trust, transparency, and perceived safety have become critical to maintaining repeat patronage [6].

This shift aligns with the transformation of consumer decision-making models, which now place greater emphasis on perceived health safety, digital convenience, and service personalization. Multiple studies have documented an increased demand for contactless dining, touchless payment systems, and visible sanitation practices, reflecting heightened consumer awareness of hygiene and risk mitigation [8,9]. These evolving expectations have made health measures a critical determinant of both initial selection and continued patronage in the restaurant industry.

In response, the casual dining sector has undergone a profound operational transition. Restaurants have adopted digital platforms for ordering and reservations, enforced stricter health protocols, and restructured service flows to reduce physical interaction. As Cha and Borchgrevink [10] note, perceived safety now significantly influences revisit intentions, while Lin et al. [11] emphasize that customer confidence in health protocols enhances trust, satisfaction, and positive word-of-mouth behavior.

Despite these shifts, traditional factors, such as food quality, pricing, and brand image, continue to shape customer expectations and satisfaction. Grounded in the Theory of Planned Behavior (TPB) and Customer Experience Theory, prior studies affirm that retention is driven by a blend of perceived value and emotional connection to the brand [12,13]. However, the current literature lacks a comprehensive framework that integrates both emerging post-COVID-19 pandemic priorities and established drivers of loyalty, particularly in the context of casual dining. This study seeks to bridge that gap by developing an integrated model to understand customer retention in a food service landscape reshaped by the pandemic.

### 1.1. Research Gap

While several studies explore post-pandemic consumer preferences and others focus on traditional drivers of loyalty, few offer an integrated framework that combines both. Most models isolate health-related concerns or examine classic determinants (e.g., food quality, price) in pre-pandemic contexts. There remains limited empirical evidence on how these variables interact under the new norms shaped by COVID-19, particularly in casual dining. This study uniquely contributes by testing a comprehensive model that incorporates both established (ambiance, location, food quality, price, brand image) and emerging factors (health measures), providing a clearer understanding of how restaurant selection leads to customer retention in the new normal.

Theoretically anchored in the Service Quality (SERVQUAL) framework and Expectation Confirmation Theory (ECT), this study explores how consumer perceptions of service elements influence behavioral intentions, such as repeat patronage and loyalty. Azad and Sadeghifar [14] and Gumasing et al. [5] emphasize the mediating role of service quality in sustaining customer engagement, while Cristobal-Lobaton et al. [7] argue that consistent service excellence provides a distinct market advantage.

This study addresses this research gap by developing and empirically testing a model that includes ambiance, location, food quality, perceived price, brand image, and health measures as predictors of casual restaurant selection, and further links selection and health measures to customer retention. Grounded in behavioral and service quality theories, the study contributes to both the literature and practice by identifying the most influential drivers of loyalty in a reshaped consumer landscape.

Practically, the study provides strategic guidance for restaurant owners in designing retention-focused interventions that reflect new customer expectations, helping food businesses navigate emerging norms while fostering sustained growth. Findings are intended to support decision making in menu planning, service design, pricing, branding, and sanitation protocols—essential components for adapting to a post-pandemic economy.

### 1.2. Objectives

This study aims to examine the key factors influencing customer retention in casual dining restaurants within the context of the “new normal” following the COVID-19 pandemic. As consumer preferences have shifted toward heightened safety awareness, digital convenience, and value-driven decisions, it is essential to identify which variables most significantly shape restaurant selection and long-term customer loyalty. Specifically, the study seeks to achieve the following:

- Assess the influences of several factors, such as ambiance, location, food quality, perceived price, brand image, and health measures, on casual restaurant selection;
- Examine the direct effect of casual restaurant selection on customer retention;
- Evaluate the role of health measures, not only in restaurant selection, but also as a direct predictor of customer retention;
- Develop and validate a structural model that reflects evolving consumer behavior in the post-pandemic food service industry;
- Provide theoretical and practical insights to help food business operators develop retention-focused strategies aligned with current consumer expectations.

By achieving these objectives, the study contributes to the literature on service quality, consumer behavior, and restaurant management, while also offering actionable recommendations for industry practitioners.

### 1.3. Conceptual Framework

**Figure 1:**
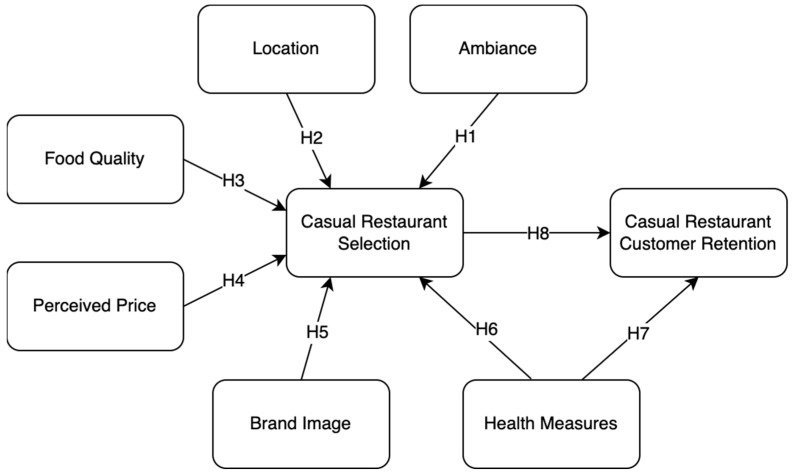
Proposed conceptual framework.

Figure 1 illustrates the conceptual framework that guides this study. The model identifies six exogenous variables—ambiance, location, food quality, perceived price, brand image, and health measures—which are hypothesized to influence casual restaurant selection and, subsequently, customer retention. These factors combine traditional service-related expectations with emergent post-COVID-19 pandemic customer concerns.

The framework is grounded in three main theoretical pillars: Service Quality Theory (SERVQUAL), Expectation Confirmation Theory (ECT), and the Theory of Planned Behavior (TPB).

From the perspective of SERVQUAL [15], customer evaluations of service quality, including tangibles (ambiance, cleanliness), reliability (brand consistency), and responsiveness (health and safety protocols), shape satisfaction and loyalty. In this study, factors such as ambiance, food quality, and perceived price are interpreted through this lens as core service attributes that drive initial selection and overall satisfaction.

Expectation Confirmation Theory [16] adds another layer, suggesting that customer loyalty results from the degree to which their pre-consumption expectations are confirmed by actual experiences. Variables such as food quality, brand image, and health measures play crucial roles in meeting customer expectations in the dining experience. If those expectations are fulfilled, especially in light of heightened concerns for health and safety, customers are more likely to return, thereby supporting retention [17].

Lastly, the framework draws from the Theory of Planned Behavior [18], which posits that behavioral intentions are shaped by attitudes, subjective norms, and perceived behavioral control. Factors like perceived price and brand image affect customers’ attitudes and confidence in making dining decisions. In the post-pandemic context, health measures have also become part of perceived behavioral control, influencing whether customers feel safe and empowered to dine out [19].

Importantly, this model integrates health measures not only as a predictor of restaurant selection, but also as a direct driver of customer retention, which is a critical addition given recent global events. As research has shown, hygiene and safety protocols are now fundamental expectations, particularly in dine-in establishments, and customers are increasingly making repeat visits based on whether these expectations are met [20].

The proposed relationship between restaurant selection and customer retention further aligns with ECT and TPB, reinforcing the notion that a positive initial experience leads to favorable behavioral intentions, such as future dining, advocacy, and loyalty.

Overall, this framework provides a comprehensive approach to understanding how both classic and contemporary factors influence customer retention in the casual dining sector. By aligning service quality variables with post-pandemic behavioral shifts, the model offers both academic relevance and practical applications for food establishments aiming to sustain customer loyalty in an evolving market.

#### 1.3.1. Ambiance

Ambiance in a restaurant refers to the overall atmosphere and mood created by physical and aesthetic elements such as lighting, music, decor, and layout. A well-designed ambiance enhances the dining experience, influencing customer emotions, satisfaction, and retention [21]. Studies have shown that ambiance plays a crucial role in a restaurant’s appeal, affecting customer decisions on whether to visit and return [21,22].

Lin and Liang [23] found that ambiance significantly influences customers’ emotional responses, overall satisfaction, and intention to revisit a restaurant. Additionally, Ozkul et al. [24] highlighted that lighting impacts customer perceptions of food and service quality, while background music can affect spending behavior and overall dining enjoyment. Kim and Moon [25] also found that restaurant decor and theme influence customer emotions and satisfaction, with modern decor eliciting higher levels of delight compared to traditional designs.

Furthermore, Ali and Amin [26] emphasized that elements such as interior design, noise control, and overall cleanliness play vital roles in shaping customers’ perceptions of service quality, which, in turn, influence dining satisfaction and long-term loyalty. Similarly, Nguyen et al. [27] found that ambient factors, such as color schemes, lighting, scent, and spatial layout, significantly affect diners’ cognitive and emotional responses, particularly in full-service and upscale casual dining environments. These environmental cues contribute to the creation of a welcoming and memorable atmosphere, which can enhance the overall dining experience and positively influence customer evaluations. Given these findings, this study hypothesizes that

**Hypothesis** **1** **(H1).** *Ambiance has a significant and direct effect on casual restaurant selection.*

#### 1.3.2. Location

The accessibility and convenience of a restaurant significantly influence customer decision making. Customers prefer restaurants that are easy to reach, in proximity to either their home or workplace, with available parking, or near public transportation options [28].

A study by Gregory & Kim [29] found that location is among the most important factors in restaurant selection, with customers favoring nearby and easily accessible establishments. Similarly, Chua et al. [30] reported that customers perceive restaurants in high-traffic or prime locations as offering better quality compared to those in less crowded areas.

In support, Ryu et al. [31] also noted that convenience of location significantly enhances perceived value and customer satisfaction, which, in turn, increases the likelihood of repeat visits. Gursoy and McCleary [32] also identified location as a top consideration for tourists selecting dining options, indicating its importance beyond local patrons. Chowdhury et al. [33] further confirmed that strategic location, including ease of access and surrounding infrastructure, contributes positively to restaurant competitiveness and customer retention. From a customer behavior perspective, a convenient location reduces time and effort, making it more likely for people to choose a restaurant. The easier it is to reach, the more attractive it becomes for both new and returning customers. Given these findings, this study hypothesizes that

**Hypothesis** **2** **(H2).** *Location has a significant and direct effect on casual restaurant selection.*

#### 1.3.3. Food Quality

Food quality encompasses various attributes, such as taste, freshness, presentation, and nutritional value, all of which influence customer satisfaction and restaurant selection [34]. High food quality has been linked to increased customer satisfaction, perceived value, and willingness to pay a premium [35]. Ryu et al. [36] found that food quality enhances a restaurant’s image and reputation, increasing the likelihood of customer selection. Similarly, Uddin [37] identified food quality as a primary driver of customer loyalty in the fast-food industry.

Furthermore, Namkung and Jang [38] highlighted that food quality significantly influences customer satisfaction and behavioral intentions in casual dining. Sulek and Hensley [39] emphasized that food quality is one of the most decisive factors in shaping overall dining satisfaction. Han and Ryu [40] also demonstrated that food quality positively affects perceived value and customer loyalty across full-service restaurant settings.

From a logical standpoint, food is the core product in any restaurant. If the taste, freshness, and presentation meet or exceed customer expectations, they are more likely to enjoy the experience, feel they received good value, and come back again. In casual dining, where price and atmosphere are more moderate, the quality of the food becomes a key reason for choosing and revisiting a restaurant. Given these findings, this study hypothesizes that

**Hypothesis** **3** **(H3).** *Food quality has a significant and direct effect on casual restaurant selection.*

#### 1.3.4. Perceived Price

Perceived price refers to customers’ assessments of whether a product or service is worth its cost [41]. Ha & Perks [42] found that customers who perceive fair pricing tend to be more satisfied with their purchase decisions.

In the restaurant industry, Ryu and Han [21] established that price perception directly affects customer loyalty, with reasonably priced restaurants enjoying higher customer retention. Jin et al. [43] further confirmed that competitive pricing enhances customer satisfaction and differentiates restaurants from competitors. Bei and Chiao [30] emphasized that perceived price fairness positively impacts customer satisfaction, increasing the likelihood of repeat visits.

In addition, Kuo et al. [44] demonstrated that price fairness significantly influences customers’ post-purchase satisfaction and loyalty in the hospitality sector. Ali and Bhasin [45] also noted that perceived price plays a key role in shaping customers’ perceived value and repurchase intentions, particularly when tied to service quality and brand reputation. Importantly, consumer income levels can shape how price is perceived; what is affordable or fair to one customer segment may not be to another, making income a significant factor in price perception and judgements.

Logically, customers want to feel that the price they paid matches the quality and service they received. If they believe they are receiving good value, they are more likely to come back. It is also important to note that people judge price differently based on their income, in that what feels affordable to one person might feel expensive to another. This means that customer income can influence how price is perceived, which, in turn, affects satisfaction and loyalty. Given these findings, this study hypothesizes that

**Hypothesis** **4** **(H4).** *Perceived price has a significant and direct effect on casual restaurant selection.*

#### 1.3.5. Brand Image

Brand image refers to customers’ perceptions of a restaurant’s reputation, identity, and overall experience. A strong brand image fosters trust, loyalty, and repeat business, while a negative perception can deter potential customers [46].

A study by Nam et al. [47] found that brand image is positively associated with customer satisfaction and loyalty, leading to increased restaurant patronage. Similarly, Koufie and Kesa [48] reported that millennials are more inclined to select restaurants based on brand image compared to older generations. Chen [49] further confirmed that a favorable brand image strongly predicts customer retention and word-of-mouth recommendations.

In line with this, Wu [50] noted that brand image significantly influences customer expectations and satisfaction in the restaurant industry. Kim et al. [51] emphasized that brand image positively affects trust, which mediates customer loyalty in service settings. Surucu et al. [52] also found that a well-positioned brand image enhances emotional attachment and revisit intention, particularly in highly competitive hospitality markets.

From a logical point of view, customers often rely on a brand’s reputation when deciding where to eat. A positive brand image gives people confidence that they will have a good experience. It also makes the restaurant more memorable and helps build an emotional connection with customers over time. Given these findings, this study hypothesizes that

**Hypothesis** **5** **(H5).** *Brand image has a significant and direct effect on casual restaurant selection.*

#### 1.3.6. Health Measures

Health and safety measures have become a key determinant of customer preferences, particularly in the post-COVID-19 pandemic restaurant industry [53]. Customers are more likely to patronize restaurants that prioritize hygiene and safety.

Ma et al. [54] found that perceived safety significantly influences customer satisfaction and revisit intentions, particularly during health crises like COVID-19. Yasami et al. [55] identified health and safety as major factors in restaurant selection, with customers favoring establishments that implement strict hygiene measures. Zibarzani et al. [56] also established that perceived safety, coupled with high service quality, enhances customer retention.

Furthermore, Pradana et al. [57] emphasized that customer perceptions of cleanliness and compliance with health regulations significantly impact return intentions in dine-in settings. Gunaydin [58] observed that COVID-19-related safety practices, such as contactless menus and frequent sanitization, improve customer trust and loyalty. Jeong et al. [19] similarly concluded that perceived hygiene transparency is a critical factor influencing positive word-of-mouth and dining satisfaction in the post-pandemic era.

From a practical point of view, this means that health and safety are no longer just background concerns, but are now central to how customers choose where to eat and whether they come back. Restaurants that show they care about customer safety are more likely to build trust and retain loyal customers in the post-pandemic world. Given these findings, this study hypothesizes that

**Hypothesis** **6** **(H6).** *Health measures have a significant and direct effect on casual restaurant selection.*

**Hypothesis** **7** **(H7).** *Health measures have a significant and direct effect on casual restaurant customer retention.*

#### 1.3.7. Casual Restaurant Selection and Customer Retention

Restaurant selection plays a crucial role in determining customer retention. Customers who have positive dining experiences are more likely to return and recommend the restaurant to others [59]. Giovanni et al. [60] found that customer satisfaction significantly influences retention and loyalty in the restaurant industry. Al-Tit [13] also observed that food quality, service quality, and ambiance are strong predictors of repeat patronage. In a study conducted by Tangtatswas et al. [61], researchers found that customer satisfaction with food quality, service, and pricing positively impacts retention in casual dining restaurants.

Additionally, Priyo et al. [62] demonstrated that experiential value and service excellence in restaurant selection enhance customer engagement and loyalty. Min and Min [63] emphasized the importance of initial restaurant choice in setting long-term customer expectations and behavioral intentions. Reibstein [64] also argued that positive first-time dining experiences significantly raise the likelihood of continued patronage and word-of-mouth endorsement.

This suggests that the initial decision to choose a restaurant is more than just a one-time action, as it can shape long-term customer behavior. If customers feel satisfied with their restaurant choice, they are more likely to return, stay loyal, and spread positive feedback. Thus, improving the factors that influence restaurant selection is a key step in strengthening customer retention. Given these findings, this study hypothesizes that

**Hypothesis** **8** **(H8).** *Casual restaurant selection has a significant and direct effect on casual restaurant customer retention.*

## 2. Methodology

### 2.1. Participants

**Figure 2:**
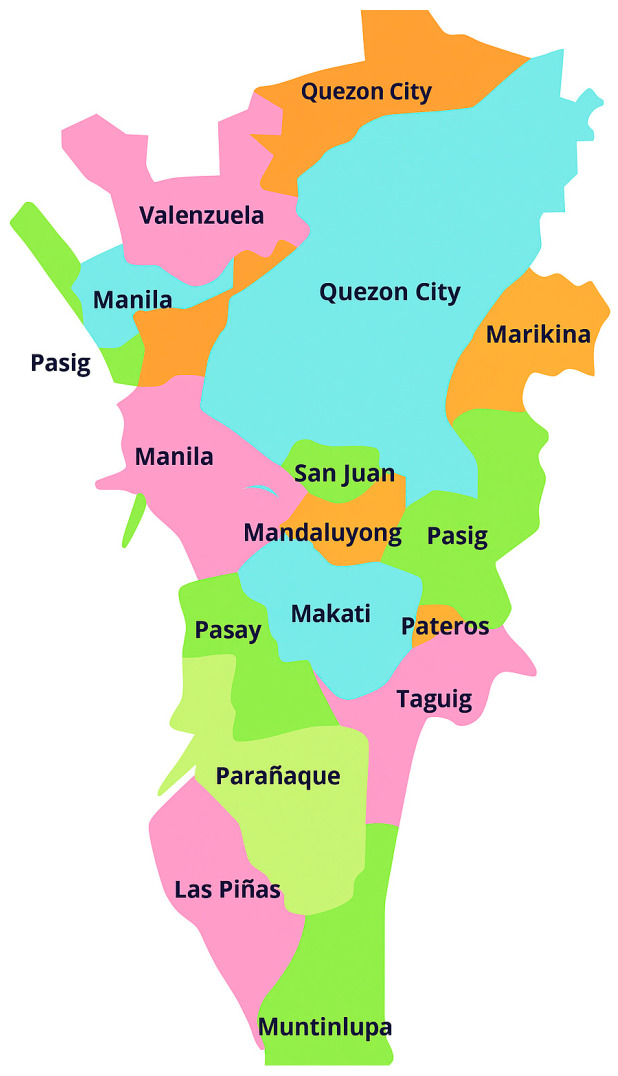
Map of the National Capital Region (NCR) Adapted from Ref. [66] 2017, Frumencio Co.

The participants of this study were customers and patrons of casual restaurants aged 18 years and above, residing within the National Capital Region (NCR), Philippines. Figure 2 presents the map of the NCR, which consists of 16 cities and one municipality, including major urban centers such as Quezon City, Manila, Makati, Pasig, Taguig, and Mandaluyong. This region was selected as the study location due to its dense population, high urbanization, and concentration of casual dining establishments catering to diverse socioeconomic groups. The NCR serves as the economic, political, and cultural hub of the country [65], making it an ideal setting for understanding consumer behaviors in the food service industry. The region’s varied commercial zones—from central business districts like Makati and Bonifacio Global City to more residential and mixed-use areas—ensure a broad representation of customer preferences and spending capacities.

Casual restaurants, as defined in this study, are dining establishments that offer a relaxed atmosphere, simple yet comfortable decor, and a moderately priced menu accessible to the general public. These establishments strike a balance between affordability and a pleasant dining experience, setting them apart from fast-food chains that emphasize speed and convenience, and from fine dining restaurants that cater to premium markets. Their inclusive appeal makes them a strategic focus for analyzing customer retention in a post-COVID-19 pandemic context.

To maintain the study’s focus, fast-food chains and fine dining restaurants were excluded from the research. Fast-food chains primarily emphasize quick service and affordability, with limited emphasis on ambiance and full-service dining, whereas fine dining establishments cater to a more exclusive clientele with premium pricing and a highly formal setting. Additionally, businesses outside of the food industry were excluded, ensuring that the findings remain specific to the casual dining sector.

### 2.2. Sample Size Determinantion

To ensure the representativeness and statistical adequacy of the results, the sample size for this study was determined based on Partial Least Squares Structural Equation Modeling (PLS-SEM) requirements, which guided both data collection and model validation processes.

A minimum sample size threshold was computed using G*Power 3.1, a commonly used statistical power analysis tool, following the study of Jhantasana [67]. For an anticipated effect size (f^2^) of 0.15 (medium), a power level of 0.80, and a significance level (α) of 0.05, with 8 predictors in the most complex regression equation, the minimum required sample size was 109.

However, to strengthen generalizability and address potential data issues, such as missing values or non-normality, a larger sample was collected. The final sample consisted of 336 respondents, which far exceeds the minimum requirement and meets the widely cited “10-times rule” for PLS-SEM suggested by Wagner and Grimm [68].

The sample was also demographically diverse, reflecting the population of casual restaurant customers in the NCR, Philippines, where a large concentration of food establishments operates. This ensures that the findings are both statistically robust and contextually representative of the target consumer market.

### 2.3. Data Gathering

A quantitative research design was employed to systematically examine the factors influencing casual restaurant selection and customer retention. Data were collected using an online survey questionnaire, which was designed to capture participants’ perceptions and experiences related to ambiance, location, food quality, perceived price, brand image, and health measures—all of which influence their restaurant selection and retention decisions.

The survey was distributed through online platforms, social media, and food-related groups, targeting individuals who had dined at casual restaurants within the NCR in the past six months. A purposive sampling method was utilized to ensure that respondents met the study’s following criteria: must be 18 years or older, must have dined at a casual restaurant within the NCR in the last six months, and must not be exclusively fast-food or fine dining customers.

The questionnaire was structured into several sections, including demographic information, dining preferences, and Likert scale questions measuring perceptions of the selected variables. Respondents were asked to rate their experiences and satisfaction levels regarding the identified factors, providing insights into their decision-making processes when selecting and returning to a casual dining establishment.

To enhance the validity and reliability of the data, a pilot test was conducted with a small group of participants prior to full-scale distribution. The feedback from this pilot test helped refine question clarity and ensure alignment with research objectives.

Ethical considerations were strictly observed throughout the data collection process. Participants were informed about the voluntary nature of their participation, data confidentiality, and anonymity. Informed consent was obtained before proceeding with the survey, ensuring compliance with research ethics and best practices.

### 2.4. Instrumentation

Data for this study were collected using an online survey questionnaire administered via Google Forms. The survey was designed to systematically examine the key factors influencing customer satisfaction and retention in casual dining restaurants.

The questionnaire aimed to gather insights on how ambiance, location, food quality, perceived price, brand image, and health measures affect customers’ dining experiences and their likelihood of returning to a restaurant. By assessing these factors, the study seeks to identify their impacts on the overall success and sustainability of casual dining establishments.

The survey consisted of multiple sections, including demographic information such as age, gender, frequency of dining at casual restaurants, and preferred dining preferences; the perception of key factors, using 5-Point Likert scale questions, with answers ranging from (1) strongly disagree to (5) strongly agree, measuring customer satisfaction and perceived importance of ambiance, location, food quality, perceived price, brand image, and health measures; and, lastly, customer retention indicators, consisting of questions assessing revisit intention, willingness to recommend, and overall dining satisfaction.

The data collected from the survey provide quantifiable insights into customer preferences and behaviors, enabling restaurants to develop strategies to enhance customer retention and operational success. The measurement items for the survey questionnaire are presented in Table S1.

### 2.5. Data Analysis

This study used Partial Least Squares Structural Equation Modeling (PLS-SEM) through SmartPLS version 3.3.3 to analyze the relationships among the variables. PLS-SEM was selected because it is suitable for studies that aim to predict key outcomes, like customer retention, especially when models involve many variables and the data may not follow a normal distribution [69].

The method works well even with moderate sample sizes, such as the 336 respondents in this study. It also allows researchers to check both the measurement model (to see if the survey items are reliable and valid) and the structural model (to test the research hypotheses). Since this study focuses on understanding behavior in a changing environment and includes multiple constructs like food quality, health measures, and brand image, PLS-SEM was the most appropriate tool for the analysis.

The measurement model evaluation assesses internal consistency reliability using Cronbach’s Alpha and Composite Reliability (CR), convergent validity through Average Variance Extracted (AVE), and discriminant validity via the Fornell–Larcker Criterion and Heterotrait–Monotrait (HTMT) Ratio. Once validated, the structural model was tested by analyzing path coefficients (β values), coefficient of determination (R^2^), effect size (f^2^), and predictive relevance (Q^2^). A bootstrapping procedure with 5000 resamples was conducted to determine the statistical significance of path coefficients.

The results confirmed whether the hypothesized factors—ambiance, location, food quality, perceived price, brand image, and health measures—significantly influence restaurant selection and customer retention. Findings provide actionable insights for restaurant owners, guiding strategies to enhance customer experience, strengthen loyalty, and ensure long-term business success in the evolving “new normal” dining landscape.

## 3. Results

### 3.1. Demographic Profile of Respondents

A total of 336 respondents participated in the survey. The gender distribution indicates a slightly higher number of female respondents (53.6%) compared to male respondents (46.4%). In terms of age, the majority of respondents belong to the 18–24 age group (80.4%), followed by smaller percentages in older age brackets, with no respondents aged 65 and above.

Regarding educational background, the largest proportion of respondents hold a Bachelor’s Degree (74.5%), followed by those with Upper Secondary Education (18.2%). A smaller percentage of respondents reported having a Doctorate Degree (1.8%) or being College Undergraduates (1.8%). In terms of occupational status, the majority are students (80.4%), followed by those who are employed (17.9%), and a small percentage who are self-employed (1.8%). Notably, no respondents reported being unemployed.

The monthly income or allowance distribution shows that the largest group falls under the “Under PHP 10,000 or USD 176.30” category (41.1%), followed by those earning between PHP 10,000 or USD 176.30–PHP 20,000 or USD 355.13 (33.9%). A smaller percentage of respondents reported incomes above PHP 50,000 or USD 878.98 (7.1%).

Overall, the demographic profile provides insights into the characteristics of the respondents, highlighting that the survey sample primarily consists of young, educated individuals, predominantly students, with moderate income levels—a key consumer group for casual dining establishments.

### 3.2. Result of Pilot Testing

To ensure the clarity, validity, and reliability of the questionnaire, a pilot test was conducted, involving 30 respondents who fit the target demographic (casual restaurant customers aged 18 and above within the NCR). The primary aim was to refine the survey instrument and confirm the internal consistency of the measurement items prior to full-scale distribution.

Validity was preliminarily assessed through item-total correlation and expert feedback. All items showed acceptable item-total correlations, above the 0.30 threshold, indicating strong individual contributions to their respective constructs. Content validity was further supported through expert review, ensuring alignment with the theoretical constructs identified in the study framework (e.g., ambiance, food quality, perceived price, brand image, health measures, and customer retention).

Reliability was evaluated using Cronbach’s Alpha. The results, as shown in Table 1, demonstrated strong internal consistency across all constructs, with alpha coefficients ranging from 0.78 to 0.89, exceeding the commonly accepted minimum threshold of 0.70.

Based on these pilot test results, no major revisions were required. Minor adjustments were made to wording for clarity, and the instrument was deemed suitable for full-scale administration.

### 3.3. Result of Initial SEM Analysis

**Figure 3:**
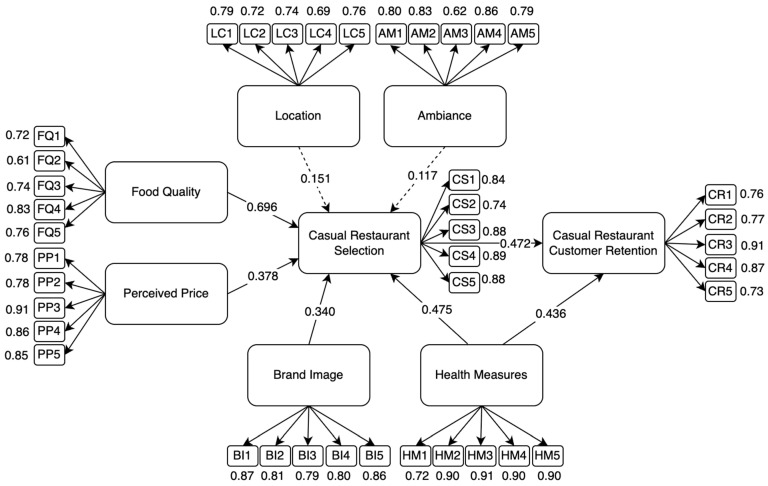
Initial SEM Framework.

The initial PLS-SEM was developed to analyze the factors influencing customer retention in casual dining restaurants after the COVID-19 pandemic. The graphical representation of the model is shown in the Figure 3, below, illustrating the relationships between key variables. The model consists of eight latent variables and a total of 40 observed indicators, representing the measured constructs.

The initial model was assessed using PLS-SEM to evaluate both the measurement model (validity and reliability of constructs) and the structural model (hypothesized relationships). Preliminary results from the measurement model indicate that all constructs met the minimum reliability and validity thresholds, confirming the appropriateness of the selected indicators. The structural model analysis provides insights into the strength and significance of the relationships between ambiance, location, food quality, perceived price, brand image, health measures, casual restaurant selection, and customer retention.

Further refinement and model adjustments were conducted to enhance predictive accuracy and ensure a robust evaluation of the hypothesized relationships. The results from this initial SEM model serve as the foundation for testing the direct and indirect effects of the identified factors on customer retention.

The validity and reliability analyses of the collected data using the proposed model are presented in Table 2. To assess the internal consistency of the variables, Cronbach’s Alpha (α) was used to determine the reliability and significance of each construct within the model. Additionally, CR and AVE were employed to evaluate the reliability and validity of the measured variables. Table 2 summarizes the results, showing that all constructs exceeded the accepted thresholds (α > 0.70, CR > 0.70, AVE > 0.50), confirming the reliability and validity of the measurement model.

CR ensures that the constructs consistently measure the intended factors, while AVE assesses the amount of variance captured by a construct relative to the variance due to measurement error. AVE is calculated as the grand mean of the squared loadings of the indicators associated with each construct, providing insight into how well the indicators represent the underlying variable. Convergent validity was also examined to determine how strongly each construct correlates with its indicators, ensuring that all items effectively measure their respective latent variables.

The results confirm that the measurement model meets the required validity and reliability thresholds. CR values exceeded the recommended threshold of 0.70, and AVE values were all above the 0.50 benchmark, indicating good internal consistency and convergent validity. All factor loadings were also greater than 0.70, further supporting the robustness of the constructs for structural model evaluation.

Discriminant validity was assessed using the Fornell–Larcker criterion and the Heterotrait–Monotrait (HTMT) Ratio of correlation to ensure that each construct is distinct from others within the model. This validation process confirms that the constructs exhibit significant correlations with their respective indicators while maintaining sufficient differentiation from other variables, thereby strengthening the measurement model’s credibility.

The results, presented in Table 3 and Table 4, indicate that all values fall within the expected thresholds, demonstrating strong reliability and convergent validity. Table 3 shows that the square roots of AVE for each construct are greater than the inter-construct correlations, confirming discriminant validity based on the Fornell–Larcker criterion. Table 4 presents the HTMT ratios, all of which are below the 0.90 threshold, further supporting discriminant validity. These findings confirm that the constructs are well-defined and effectively measured, ensuring the overall acceptability and robustness of the model for further structural analysis.

Before interpreting the path coefficients, multicollinearity was assessed using Variance Inflation Factor (VIF) values. All predictor variables reported VIF values below the recommended threshold of 5.0 [70], confirming the absence of multicollinearity issues. Table 5 provides a summary of the VIF values for each construct in the structural model, all of which fall within acceptable limits, ensuring that the predictors do not distort the regression estimates.

After this, the PLS-SEM analysis was conducted to test the proposed hypotheses, with the results presented in Table 6. The findings reveal that casual restaurant retention is significantly influenced by casual restaurant selection (β = 0.475, *p* < 0.001), indicating that customers who positively perceive a restaurant’s selection criteria are more likely to return. Table 6 summarizes the path coefficients, *p*-values, and significance levels for each hypothesized relationship, highlighting which factors have meaningful impacts on restaurant selection and customer retention.

Among the key factors, health measures significantly impact both casual restaurant selection (β = 0.477, *p* = 0.001) and customer retention (β = 0.436, *p* = 0.002), highlighting the heightened importance of safety and hygiene in the post-pandemic dining environment. Additionally, brand image (β = 0.341, *p* = 0.035), perceived price (β = 0.378, *p* = 0.002), and food quality (β = 0.698, *p* < 0.001) were found to have significant influences on casual restaurant selection, highlighting their role in shaping customer preferences.

Conversely, location (β = 0.151, *p* = 0.273) and ambiance (β = 0.117, *p* = 0.415) were found to be insignificant in influencing casual restaurant selection after the COVID-19 pandemic. This suggests that, while traditionally important, these factors may have diminished relevance in customer decision making compared to health measures, food quality, pricing, and brand perception.

The model fit analysis confirmed the overall validity and robustness of the proposed model. As shown in Table 7, all model fit indices and parameter estimates met or exceeded the established minimum thresholds, such as SRMR and R^2^ values, indicating a good fit between the model and the observed data. These results validate the suitability of the model for assessing the factors influencing casual restaurant selection and customer retention, reinforcing its reliability for further interpretation and strategic insights.

### 3.4. Result of Final SEM Analysis

**Figure 4:**
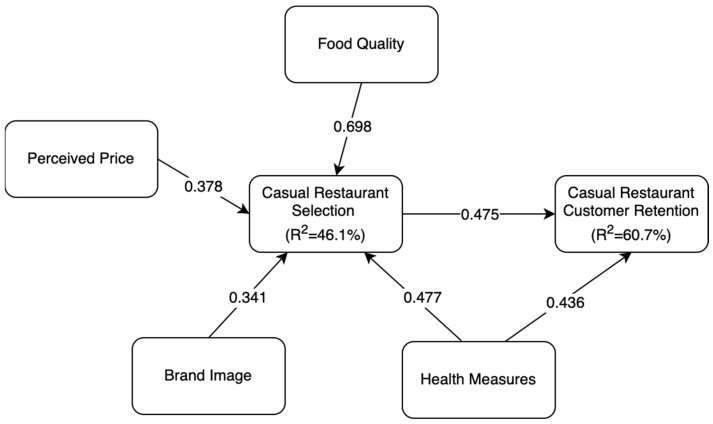
Final SEM framework.

The final Structural Equation Model (SEM), illustrated in Figure 4, below, presents the completed framework, incorporating hypothesis testing to evaluate the beta coefficients and R^2^ values of the proposed model. The results indicate that casual restaurant selection explains 60.7% of its own variance, while it contributes 46.1% to casual restaurant retention. These findings demonstrate that the model effectively captures and predicts the key factors influencing restaurant selection and customer retention, validating its robustness in understanding consumer behavior within the casual dining industry.

## 4. Interpreting Key Drivers of Customer Retention in Post-COVID-19 Pandemic Casual Dining

This study fills a research gap by empirically testing a model that links ambiance, location, food quality, perceived price, brand image, and health measures to casual restaurant selection, and further connects these factors, particularly health measures and selection, to customer retention. Drawing from behavioral and service quality theories, the findings highlight key drivers of customer loyalty in a post-COVID-19 pandemic context. Practically, the study offers actionable insights for restaurant managers to align their strategies with evolving consumer expectations, informing decisions on menu design, pricing, branding, service delivery, and hygiene protocols to foster long-term retention and business growth.

The results of this study reveal that food quality has the most significant impact on casual restaurant selection (β = 0.698, *p* < 0.05), thereby confirming H3. This finding suggests that customers who have a positive experience with food taste, freshness, presentation, and overall quality are more likely to return for repeat visits. This aligns with Uddin [37], who identified food quality and taste as critical drivers of customer loyalty in the fast-food industry. Similarly, Ryu et al. [31] found that food quality significantly shapes a restaurant’s perceived image and reputation, directly influencing customers’ choices. Given that food quality is central to customer satisfaction and retention, casual dining establishments should prioritize consistent food standards to enhance their market competitiveness and build long-term customer loyalty.

Notably, post-pandemic studies have also confirmed the heightened role of food quality in consumer behavior. For instance, Jeong et al. [19] found that, in the aftermath of COVID-19, food safety and freshness have become even more critical to customers’ dining choices, especially in dine-in settings. Wu et al. [73] also reported that consumers now associate high food quality not only with taste, but also with trustworthiness and safety, which reinforces the importance of consistent standards.

Additionally, the findings reveal that perceived price (β = 0.378, *p* < 0.02) and brand image (β = 0.341, *p* < 0.035) significantly influence casual restaurant selection, confirming H4 and H5. This indicates that customers who perceive pricing as fair and reasonable are more likely to return and recommend the restaurant to others. This supports prior post-COVID-19 research by da Silva [74], who found that fair pricing enhances customer loyalty, and by Hride et al. [75], who showed that competitive pricing contributes to satisfaction and repeat patronage. Yang et al. [76] likewise concluded that perceived price fairness positively influences overall customer experience and retention.

Beyond satisfaction and loyalty, perceived price also shapes consumers’ social identities and perceived statuses. Dining at certain price points can convey prestige or exclusivity. Balabanis and Stathopoulou [77] noted that consumers often associate price with social status and self-image, suggesting that price perception extends beyond utility. In the context of post-pandemic recovery, Ahmed et al. [78] emphasized that consumers became more conscious of value for money, while still valuing experiences that reinforce social distinction. Similarly, Taneja et al. [79] found that post-COVID diners were more likely to choose restaurants that aligned with their self-concept and social aspirations, even if prices were higher.

Similarly, brand image plays a pivotal role in restaurant selection and customer loyalty. Nam et al. [47] and Kaur and Soch [80] found that a strong brand image fosters trust, customer satisfaction, and repeat visits, particularly among younger consumers. Jin et al. [43] also emphasized that a positive emotional connection to a brand increases customer loyalty. Recent post-pandemic studies, such as those by Sharma and Romero [81] and Kim et al. [82], found that brand reputation became even more critical as consumers sought familiar and reliable establishments during uncertain times. These studies suggest that, after the COVID-19 pandemic, consumers are more brand-conscious, associating trusted brands with safety, consistency, and emotional reassurance. Therefore, restaurants should strategically manage their brand identity, ensuring consistent service quality, fostering positive customer experiences, and employing targeted marketing, to reinforce brand perception and sustain customer retention.

Another critical factor influencing both casual restaurant selection (β = 0.477, *p* = 0.001) and customer retention (β = 0.436, *p* = 0.002) is health measures, supporting H6 and H7. This highlights the increasing importance of safety and hygiene standards in post-pandemic consumer behavior. Mannan et al. [83] confirmed that customers are more likely to revisit restaurants they perceive as safe, while Byrd et al. [84] emphasized that health and safety concerns became decisive factors in dining choices during and after the COVID-19 crisis. Recent studies, such as those by Tuncer et al. [85] and Ababneh et al. [86], further support these findings by highlighting that perceived safety strongly correlates with customer satisfaction and behavioral intentions in the food service industry. This study reinforces the notion that visible and reliable health measures not only influence immediate customer choices, but also foster long-term trust and loyalty. Additionally, Gumasing et al. [87] observed that, when health protocols are complemented by high service quality, customers are more likely to develop strong retention behavior. In light of this, restaurants must implement and sustain robust health and safety practices to meet heightened consumer expectations and remain competitive in the evolving dining landscape.

The study also confirms that casual restaurant selection significantly affects customer retention (β = 0.475, *p* < 0.05), supporting H8. This finding aligns with Al-Tit [13], who found that customers who enjoy their dining experience are more inclined to return and recommend the restaurant to others. Similarly, Lee et al. [88] observed that customer satisfaction and restaurant choice significantly impact retention and loyalty. Post-pandemic studies, such as those by Wen and Liu-Lastres [89] and Bichler et al. [90], further emphasize that, in the context of increased consumer caution, a memorable first dining experience, marked by excellent food, attentive service, and a welcoming atmosphere, has become even more crucial in fostering repeat visits. This aligns with Min & Min [63], who stressed that consistency, responsiveness to customer feedback, and distinctive offerings are essential in retaining customers and ensuring long-term business sustainability. In sum, restaurant operators must recognize that the initial experience is a gateway to building enduring relationships with their patrons in the post-pandemic dining landscape.

Contrary to expectations, ambiance was found to have no significant effect on casual restaurant selection (β = 0.117, *p* = 0.415), resulting in the rejection of H1. This suggests that, in the post-pandemic dining environment, customers may prioritize factors such as food quality, pricing, and safety over the aesthetic or atmospheric elements of a restaurant. Bichler et al. [90] similarly observed that diners are often willing to overlook ambiance if the restaurant delivers high-quality food and attentive service. Makwena [22] also found that, while ambiance contributes to the overall dining experience, it is frequently outweighed by more functional considerations like affordability and taste. Recent post-pandemic studies, such as those by Ma et al. [54] and Spence et al. [91], support this shift, noting that consumers are more value-driven and cautious, placing greater emphasis on tangible benefits over experiential factors. In this context, ambiance may serve more as a supporting feature, rather than a decisive factor in restaurant selection. For casual dining establishments, this highlights the importance of focusing on core service attributes while maintaining a pleasant, but not necessarily elaborate, environment.

Additionally, location was found to have an insignificant influence on casual restaurant selection (β = 0.151, *p* = 0.273), leading to the rejection of H2. This suggests that customers are willing to travel for a superior dining experience, rather than selecting a restaurant solely based on convenience. Njite et al. [92] and Hwang et al. [93] found that customers often prioritize food preference over proximity. In the post-pandemic context, this trend has become even more pronounced. The widespread adoption of online food delivery services has diminished the importance of physical location for many consumers, allowing them to access their preferred restaurants regardless of distance [94]. Similarly, Min and Min [68] noted that improvements in transportation options, such as ridesharing and increased mobility, have further reduced the inconvenience associated with traveling to dine out. Recent studies, including those by Meethavornkul et al. [95] and Aytac et al. [96], also observed that post-COVID consumers value perceived quality, hygiene, and brand trust more than geographical closeness, especially when health and satisfaction are at stake. These findings collectively indicate that location, once a primary driver of casual restaurant choice, now plays a more secondary role in the evolving dining landscape.

The findings of this study highlight that food quality, health measures, perceived price, brand image, and restaurant selection are the primary drivers of customer retention in casual dining restaurants. While ambiance and location remain secondary considerations, ensuring high food quality, maintaining strong health and safety standards, offering fair pricing, and building a strong brand identity are critical for attracting and retaining customers. These insights provide valuable guidance for restaurant owners and managers seeking to enhance their customer retention strategies in the evolving post-pandemic dining landscape.

Based on the validated hypotheses, this study proposes a strategic intervention framework aimed at enhancing customer retention in casual dining restaurants. Table 8 presents the intervention strategies, which are aligned with the key determinants identified through the PLS-SEM analysis, specifically food quality, health measures, perceived price, brand image, and overall customer experience. These strategies offer actionable recommendations for restaurant managers to address customer expectations and sustain loyalty in the post-pandemic dining landscape.

## 5. Concluding Insights on Customer Retention After the COVID-19 Pandemic

This study explored the key factors influencing customer retention in casual dining restaurants within the new normal, using PLS-SEM to examine the relationships between food quality, health measures, perceived price, brand image, ambiance, and location. The findings confirm that food quality has the most significant impact on restaurant selection, highlighting the importance of taste, freshness, and presentation in fostering customer loyalty. Health measures also significantly influence both restaurant selection and customer retention, reflecting the heightened consumer focus on safety and hygiene in post-pandemic dining behavior.

Perceived price and brand image were also found to meaningfully affect restaurant selection, indicating that affordability and a strong brand reputation enhance a restaurant’s ability to attract and retain customers. Furthermore, casual restaurant selection directly influences customer retention, supporting the view that a positive initial dining experience leads to repeat visits and favorable word-of-mouth.

While the study offers valuable insights into customer retention in Philippine casual dining, its findings may not be fully generalizable to other cultural or geographic settings. The results are based on a specific consumer group within the National Capital Region (NCR), where digital adoption and health awareness may differ from rural or international contexts. Furthermore, the study focused solely on casual dining; thus, the conclusions may not directly apply to fast-food or fine dining segments. Future research could replicate this model in other regions or countries to test for cross-cultural applicability and sectoral differences in customer retention strategies.

Theoretical implications of the study include the integration of health measures into consumer behavior and service quality models, particularly under crisis-affected scenarios, an area previously underexplored. Practically, the study provides restaurant operators with clear guidance. Investing in food quality, maintaining visible health protocols, offering fair pricing, and building a reputable brand can strengthen customer retention strategies.

Limitations include the geographic focus on the NCR of the Philippines and the use of a cross-sectional design. Future research may expand to other regions or use longitudinal data to track evolving behaviors. Additionally, studies can explore digital transformation, sustainability, and emotional engagement as emerging drivers of customer loyalty in the food service industry.

### 5.1. Practical and Managerial Implications

The findings of this study provide valuable insights for casual dining restaurant owners, managers, and industry stakeholders seeking to enhance customer retention and long-term business sustainability. By understanding the key factors influencing restaurant selection and customer loyalty, businesses can implement targeted strategies to improve service quality, optimize customer experience, and strengthen their competitive positioning in the market.

Since food quality (β = 0.698, *p* < 0.05) emerged as the most significant factor influencing restaurant selection, casual dining establishments must prioritize ingredient freshness, consistency in taste, portion control, and presentation to enhance customer satisfaction. Regular quality audits, chef training programs, and customer feedback mechanisms should be implemented to maintain high food standards and improve perceived value.

With health measures significantly impacting restaurant selection (β = 0.477, *p* = 0.001) and retention (β = 0.436, *p* = 0.002), restaurants must continuously enforce stringent hygiene and sanitation protocols. Visible cleanliness efforts, contactless payment options, well-sanitized dining areas, and staff compliance with hygiene regulations can enhance customer trust. Moreover, digital transparency—such as posting health certifications or sanitation procedures on social media—can reinforce a restaurant’s commitment to customer safety.

The significant influence of perceived price (β = 0.378, *p* < 0.02) suggests that restaurants should carefully structure their pricing strategy to balance affordability and perceived value. Managers should consider loyalty programs, bundle promotions, and discounts for repeat customers to encourage long-term patronage. Flexible menu pricing that caters to different spending preferences can also widen the target market without compromising profitability.

With brand image (β = 0.341, *p* < 0.035) proving to be a key determinant in restaurant selection, casual dining establishments should invest in brand development, customer engagement, and reputation management. Consistent branding, engaging digital marketing, and an active social media presence can help restaurants build a strong emotional connection with customers. Encouraging user-generated content, customer testimonials, and influencer collaborations can further enhance brand credibility and loyalty.

The study found ambiance (β = 0.117, *p* = 0.415) and location (β = 0.151, *p* = 0.273) to be insignificant in restaurant selection, indicating that customers prioritize food quality, pricing, and safety over aesthetics and proximity. Instead of heavy investments in decor or prime locations, restaurants should focus on service efficiency, digital accessibility (e.g., online reservations and food delivery services), and operational excellence to enhance customer satisfaction and retention.

Since casual restaurant selection directly impacts customer retention (β = 0.475, *p* < 0.05), first-time customer experience plays a pivotal role in encouraging repeat visits. Managers should ensure that seamless service, minimal waiting times, personalized interactions, and consistent food quality create a memorable dining experience. Implementing customer relationship management (CRM) tools, post-dining surveys, and real-time service feedback can help refine operations and improve retention strategies.

### 5.2. Theoretical Implications

This study contributes to the theoretical understanding of customer retention in the casual dining industry by expanding on existing models related to consumer behavior, service quality, and restaurant selection in the new normal dining environment. The findings offer significant implications for theories related to customer satisfaction, loyalty, and decision-making processes, particularly in the post-COVID-19 pandemic era, where health measures and digital transformation have reshaped consumer expectations.

First, the study reinforces and extends the SERVQUAL model [15] by confirming that food quality, perceived price, and brand image significantly impact restaurant selection, while health measures play a crucial role in customer retention. Traditional service quality dimensions, such as tangibles (ambiance) and convenience (location), were found to be less significant, suggesting that, in the new normal, safety, hygiene, and perceived value have become more dominant factors in customer decision making.

Second, the significant influence of food quality and perceived price on restaurant selection aligns with Expectation Confirmation Theory [16], which suggests that customer satisfaction and retention occur when actual experiences meet or exceed initial expectations. This study extends ECT by demonstrating that health measures also contribute to expectation confirmation, as customers prioritize safety and hygiene in their dining choices, influencing their likelihood to return.

Third, the study supports the Theory of Planned Behavior by Azjen [18], by showing that consumer attitudes toward food quality, pricing fairness, and health safety measures directly influence their restaurant selection and retention behaviors. The findings suggest that post-pandemic consumer behavior is shaped by a stronger emphasis on perceived health risks and financial considerations, indicating a shift in behavioral intentions and decision-making patterns in the restaurant industry.

Finally, the finding that casual restaurant selection significantly influences customer retention reinforces existing theories of customer experience and brand loyalty [97]. However, it also introduces a new perspective on the importance of first-time dining experiences in influencing repeat visits. The results suggest that restaurants should not only focus on maintaining long-term customer relationships, but also ensure that first-time customers receive a high-quality experience to increase the likelihood of retention.

### 5.3. Limitations and Future Research

This study has several limitations that provide opportunities for future research. One notable limitation of this study is the demographic imbalance of the sample, with approximately 80% of respondents consisting of students, most of whom are presumed to be within the younger age bracket. While this group represents an important consumer segment for casual dining restaurants, given their active social habits and spending on food services, their preferences may not reflect those of older or working populations. As a result, the findings may be more representative of youth-driven dining behavior and less generalizable to broader demographic groups, such as middle-aged professionals or families, who may prioritize different factors, such as convenience, nutritional value, or family-oriented amenities. Future research should aim for a more balanced demographic distribution to better capture diverse consumer perspectives and enhance the applicability of findings across age groups and income levels.

Second, the study focused only on casual dining restaurants in the NCR, Philippines, limiting the generalizability of findings to other regions or restaurant types (e.g., fine dining, fast food). Future research could expand the geographical scope or compare different restaurant categories to validate the findings across diverse dining contexts.

Third, the study utilized cross-sectional data, capturing consumer behavior at a single point in time. Given the evolving nature of consumer preferences, longitudinal studies could be conducted to examine how factors influencing restaurant selection and retention change over time, especially as post-COVID-19 pandemic dining behaviors stabilize. This would allow future researchers to observe changes in consumer behavior over time, especially as health concerns and market conditions evolve beyond the pandemic. A longitudinal study could also provide deeper insights into how customer retention develops and shifts in response to long-term trends.

Fourth, the study used only quantitative data from an online survey. While this helped identify important patterns and relationships between the factors, it may not fully explain the reasons behind customer choices or behaviors. Using a mixed-methods approach, such as interviews or focus group discussions with restaurant managers or customers, could provide deeper insights and help researchers better understand the context behind the study results. Future research is encouraged to combine both survey data and personal experiences to give a more complete background of what drives customer retention in casual dining, especially in the changing post-COVID-19 pandemic environment.

Lastly, the study primarily focused on food quality, pricing, brand image, and health measures, while excluding other potential determinants, such as digital innovations (e.g., online ordering, AI-driven personalization) and sustainability practices. Future research could explore how technology adoption and environmental concerns influence customer retention in the evolving food industry landscape.

By addressing these limitations, future studies can provide a more comprehensive understanding of consumer behavior, helping restaurant businesses adapt to emerging trends and enhance long-term customer loyalty.

## Figures and Tables

**Table 1:** Result of pilot testing.

Construct	No. of Items	Cronbach’s Alpha
Ambiance	5	0.81
Location	5	0.78
Food Quality	5	0.89
Perceived Price	5	0.83
Brand Image	5	0.87
Health Measures	5	0.88
Restaurant Selection	5	0.84
Restaurant Retention	5	0.88

**Table 2:** Reliability and convergent validity results.

Construct	Items	Mean	Standard Deviation	Factor Loading (≥0.7)	Cronbach’s α (≥0.7)	Composite Reliability (≥0.7)	Average Variance Extracted (≥0.5)
Ambiance (AM)	AM1	3.51	1.03	0.793	0.796	0.809	0.550
	AM2	3.43	1.07	0.716			
	AM3	3.21	1.07	0.744			
	AM4	3.60	0.99	0.692			
	AM5	3.56	1.03	0.758			
Location (LC)	LC1	3.68	1.04	0.796	0.842	0.864	0.612
	LC2	3.54	1.09	0.829			
	LC3	3.56	1.11	0.621			
	LC4	3.85	1.11	0.855			
	LC5	3.57	1.02	0.791			
Food Quality (FQ)	FQ1	3.47	1.00	0.723	0.786	0.799	0.540
	FQ2	3.37	0.96	0.613			
	FQ3	3.46	0.94	0.740			
	FQ4	3.40	1.07	0.826			
	FQ5	3.56	1.06	0.756			
Perceived Price (PP)	PP1	3.60	1.07	0.782	0.853	0.864	0.695
	PP2	3.63	1.06	0.776			
	PP3	3.62	1.03	0.910			
	PP4	3.65	1.05	0.859			
	PP5	3.57	1.06	0.846			
Brand Image (BI)	BI1	3.30	1.06	0.865	0.835	0.842	0.667
	BI2	3.33	1.05	0.814			
	BI3	3.64	1.07	0.785			
	BI4	3.67	0.97	0.801			
	BI5	3.59	1.05	0.862			
Health Measures (HM)	HM1	3.41	1.11	0.715	0.918	0.943	0.755
	HM2	3.30	1.11	0.897			
	HM3	3.51	1.11	0.913			
	HM4	3.45	1.06	0.904			
	HM5	3.45	1.06	0.900			
Casual Restaurant Selection (CS)	CS1	3.41	1.11	0.835	0.901	0.919	0.717
	CS2	3.30	1.11	0.738			
	CS3	3.51	1.11	0.883			
	CS4	3.45	1.06	0.889			
	CS5	3.45	1.06	0.879			
Casual Restaurant Retention (CR)	CR1	3.41	1.11	0.775	0.871	0.884	0.664
	CR2	3.30	1.11	0.774			
	CR3	3.51	1.11	0.914			
	CR4	3.45	1.06	0.865			
	CR5	3.45	1.06	0.733			

**Table 3:** Discriminant validity: Fornell–Larcker criterion.

	Ambiance	Brand Image	Customer Retention	Custommer Selection	Food Quality	Location	Perecived Price	Health Measures
Ambiance	0.742							
Brand Image	0.642	0.817						
Customer Retention	0.606	0.582	0.815					
Customer Selection	0.580	0.660	0.642	0.847				
Food Quality	0.534	0.535	0.547	0.566	0.735			
Location	0.334	0.336	0.395	0.499	0.550	0.783		
Perceived Price	0.565	0.322	0.402	0.519	0.533	0.501	0.835	
Health Measures	0.411	0.490	0.563	0.601	0.412	0.427	0.575	0.869

**Table 4:** Discriminant validity: Heterotrait–Monotrait Ratio.

	Ambiance	Brand Image	Customer Retention	Custommer Selection	Food Quality	Location	Perecived Price	Health Measures
Ambiance								
Brand Image	0.775							
Customer Retention	0.718	0.682						
Customer Selection	0.652	0.738	0.697					
Food Quality	0.669	0.653	0.664	0.647				
Location	0.427	0.359	0.425	0.570	0.661			
Perceived Price	0.702	0.356	0.447	0.581	0.625	0.585		
Health Measures	0.470	0.551	0.608	0.637	0.458	0.458	0.626	0.869

**Table 5:** Variance Inflation Factor (VIF) values for predictor constructs.

Predictable Variable	VIF Value
Ambiance	1.123
Location	1.281
Food Quality	2.319
Perceived Price	1.943
Brand Image	1.887
Health Measures	1.598

**Table 6:** Hypothesis test.

Hypothesis	Path Coefficient (β)	*p*-Value	Effect Size (f^2^)	Conclusion
H1: Ambiance → Casual Restaurant Selection	0.117	0.415	0.02	Reject
H2: Location → Casual Restaurant Selection	0.151	0.273	0.03	Reject
H3: Food Quality → Casual Restaurant Selection	0.698	0.001	0.35	Do not reject
H4: Perceived Price → Casual Restaurant Selection	0.378	0.002	0.18	Do not reject
H5: Brand Image → Casual Restaurant Selection	0.341	0.035	0.12	Do not reject
H6: Health Measures → Casual Restaurant Selection	0.477	0.001	0.25	Do not reject
H7: Health Measures → Casual Restaurant Retention	0.436	0.002	0.21	Do not reject
H8: Casual Restaurant Selection → Casual Restaurant Retention	0.475	0.001	0.28	Do not reject

**Table 7:** Model fit.

Model Fit for SEM	Parameter Estimates	Minimum Cut-Off	Recommended by
SRMR	0.068	<0.08	[71,72]
(Adjusted) Chi-square/dF	4.74	<5.0	
Normal Fit Index (NFI)	0.946	>0.90	

**Table 8:** Summary of interventions.

Relationship	Summary of Results	Supporting RRL	Intervention
Food Quality → Casual Restaurant Selection	A study reveals that food quality significantly affects the perceived image and reputation of a restaurant. Customers’ perceptions of a restaurant’s quality are directly tied to the quality of its food, and this influences their choice of where to dine.	Ryu et al. [31]; Uddin [37]	Businesses in the food industry should prioritize and maintain high food quality standards to attract and retain customers, ultimately contributing to their success.
Price → Casual Restaurant Selection	It was found that there is a notable connection between price and customer loyalty. The research revealed that customers who hold a positive perception of prices are more inclined to become returning patrons and advocate for the restaurant to others.	Ryu & Han [21]; Jin et al. [43]	Offering competitive prices can effectively set restaurants apart from competitors and cultivate a favorable perception among customers. One tactic is to concentrate on offering customers value. Make sure the amount charged for the ambiance, food, and service in a casual restaurant is reasonable. Ensure that the eating experience meets the expectations of the target audience.
Brand Image → Casual Restaurant Selection	It was ascertained that a restaurant’s brand image serves as a potent predictor of customer loyalty. The research illustrates that customers who derive a positive emotional connection from a restaurant’s brand image are more likely to become repeat customers.	Jin et al. [43]; Nam et al. [47]; Kaur and Soch [80]	It is suggested to provide regular menu updates, emphasizing premium products and specialty dishes that can strengthen a casual restaurant’s brand identity. Make customer service a priority and train the team to deliver outstanding experiences. The restaurant’s ambience, atmosphere, and interior design should reflect your corporate identity to give customers a memorable, seamless dining experience.
Health Measures → Casual Restaurant Selection	It is observed that health and safety concerns were a significant factor in restaurant choice during the COVID-19 pandemic. Customers are more likely to select a restaurant they perceive as safe, and which takes appropriate health precautions. This confirms that health measures influence customers’ decisions when choosing where to dine. This highlights the importance of maintaining health and safety standards alongside excellent service to retain customers	Mannan et al. [83]	Casual restaurants should maintain a strong focus on health measures to build trust and loyalty among their customer base. Implement stringent cleaning routines and techniques, paying close attention to kitchen tools, utensils, and surfaces that are likely to be touched frequently. Utilize disinfectants that adhere to health standards. Follow food safety regulations to the extent necessary to avoid contamination. Verify correct storage practices and frequently check food expiration dates. Choose ingredients from reliable vendors who adhere to hygienic and safe procedures. When possible, take into account sustainable and local sourcing.
Health Measures → Casual Restaurant Retention	A study by Pradana et al. [57] also confirmed this finding. It was found that customers who perceive the restaurant as safe are more likely to have revisit intentions.	Byrd et al. [84]	
Casual Restaurant Selection → Casual Restaurant Retention	This proves the study from Al-tit [13], which found that individuals who find their dining experience enjoyable are more inclined to revisit the restaurant and offer recommendations to others. Furthermore, research has revealed that the choices made by customers significantly enhance customer retention within the restaurant industry.	Al-Tit [13]; Lee et al. [88]	Factors influencing restaurant selection and retention are crucial for building a loyal customer base. That entails providing excellent, delectable meals and a menu with something for everyone. The restaurant can distinguish whether it serves unusual or exceptional cuisine. It is also critical to offer options for those with various budgets and fair prices. Restaurants should have a welcoming and knowledgeable staff, as they can significantly influence how customers perceive the business. A beautiful, clean environment is also essential. It is crucial to be active online, engage with clients on social media, and request positive feedback.

## Data Availability

The original contributions presented in the study are included in the article, further inquiries can be directed to the author.

## Supplementary Materials

The following supporting information can be downloaded at: https://www.mdpi.com/article/10.3390/foods14142535/s1, Table S1: Summary of measured items [98,99,100,101,102,103,104,105,106,107,108,109,110,111].
