# Supplementary material for: Customer Retention in the Philippine Food Sector: Health Measures, Market Access, and Strategic Adaptation After the COVID-19 Pandemic
Source: Foods. 2025 Jul 19;14(14):2535. doi: 10.3390/foods14142535 (PMC12294402; doi:10.3390/foods14142535)
Supplement: Supplementary file 1 [file foods-14-02535-s001.zip › foods-3721680-supplementary.pdf]

**Table S1**. Summary of measured items.

| Indicator | Sub-indicator | Items | Reference |
|---|---|---|---|
| Ambiance | A1—Visual appeal | The interior design and decorations of the casual restaurant are visually appealing. | Sharma et al. [81]; Hyun et al. [98] |
| | A2—Furniture and fixtures | The furniture and fixtures of the casual restaurant (e.g., dining table, chair) are clean and well-maintained. | |
| | A3—Layout | The seating arrangement in the casual restaurant is comfortable and has enough space. | |
| | A4—Music | The music in the casual restaurant compliments the ambiance. | |
| | A5—Decor | The décor in the casual restaurant is visually appealing. | |
| Food quality | F1—Food presentation | The food in the casual restaurant has a nice presentation. | Ashraf et al. [99]; Abdullah et al. [100] |
| | F2—Food condition | The food in the casual restaurant is served hot. | |
| | F3—Food variety | The casual restaurant offers variety in their menu. | |
| | F4—Food freshness | The casual restaurant uses fresh ingredients for the dishes. | |
| | F5—Food preparation | The foods served in the casual restaurant are well prepared. | |
| Location | L1—Accessibility | The restaurant's location is easily accessible. | Heo & Kim [101]; Ho [102] |
| | L2—Safety | I am satisfied with the availability of food establishments that offer safe and convenient locations during the "New Normal"? | |
| | L3—Convenience | The location provides parking for customers with vehicle. | |
| | L4—Environment | The presence of other businesses or attractions near the food establishment's location enhances my overall experience and satisfaction during the "New Normal." | |
| | L5—Traffic | The traffic conditions and congestion around the food establishment's location affect my likelihood of revisiting during the "New Normal." | |
| Perceived Price | P1—Price competitiveness | The casual restaurant offers competitive pricing. | Meng [103]; Chiang and Jang [104] |
| | P2—Reasonable price | The food quality is reasonable for its price. | |
| | P3—Price fairness | The price is fair for both quality and service of the casual restaurant. | |
| | P4—Value for money | The casual restaurant provides good value for money. | |
| | P5—Price affordability | The casual restaurant provides affordable price. | |
| Brand image | BI1—Brand trustworthiness | The casual restaurant brand image is trustworthy. | Dam & Dam [105]; Tu et al. [106] |
| | BI2—Brand reliability | The casual restaurant brand image is reliable. | |

| | | | |
|---|---|---|---|
| | BI3—High-quality product/service | The casual restaurant brand is associated with high-quality product/service. | |
| | BI4—Brand recognition | The casual restaurant brand is well-known and recognized in the industry. | |
| | BI5—Brand consistency | The casual restaurant brand consistently delivers on its promises and meets customer expectations. | |
| Health Measures | HM1—Safe dining | I feel safe dining at casual restaurant during the pandemic and after the pandemic. | Alkhadim et al. [107]; Cakit et al. [108] |
| | HM2—Safety measures | The casual restaurant has taken appropriate safety measures to protect customers and staff during and after the pandemic. | |
| | HM3—Safety and health protocols | The casual restaurant follows all necessary safety and health protocols during and after the pandemic. | |
| | HM4—Sanitation and hygiene protocols | The casual restaurant follows all necessary sanitation and hygiene protocols. | |
| | HM5—Perceived safety level | I am satisfied with the casual restaurant perceived level of safety during my dining experience. | |
| Customer Selection | CS1—Food and service satisfaction | I am satisfied with both the food products and services of the casual restaurant. | Ramayah & Lee [109]; Gumasing & Ilo [110] |
| | CS2—Food and service quality | The quality of food and services at the casual restaurant is what the customers expect. | |
| | CS3—Food establishment restaurant quality | The casual restaurants succeeded in getting a better-perceived restaurant quality. | |
| | CS4—Staff attentiveness | I am satisfied with the quality of service of the casual restaurant staff. | |
| | CS5—Cleanliness and ambiance | I am satisfied with the overall cleanliness and ambiance of the casual restaurant. | |
| Customer Retention | CR1—Future dining | I would consider dining in casual restaurant in the future. | Salamah et al. [111] |
| | CR2—Loyalty | I believe that customer loyalty is crucial for the long-term success of the casual restaurant. | |
| | CR3—Strategies | The casual restaurant provides effective strategies in improving customer retention. | |
| | CR4—Customer experience | The casual restaurant invests in customer experience and satisfaction to improve customer retention. | |
| | CR5— Feedback | The casual restaurant effectively responds to negative feedback and complaints to retain customers. | |
